# Supplementary material for: Content-rich biological network constructed by mining PubMed abstracts
Source: BMC Bioinformatics. 2004 Oct 8;5:147. doi: 10.1186/1471-2105-5-147 (PMC528731; doi:10.1186/1471-2105-5-147)
Supplement: Additional File 2 — The original results of the above study (non-essential files are deleted to keep the file size under the limit set by BMC bioinformatics). [file 1471-2105-5-147-S2.bz2 › chilibotAdditionalFile2/dip10/148ID9160881E524/html/CBL_LYN.html]

 


 **CBL** and **LYN** 
  
Found 24 abstracts in PubMed, retrieved 10.  
 

 What does Google say? 
 PDF only 
| .edu only 

---

**Interactive relationship** (e.g. stimulation, inhibition, etc)

**Inhibitory relationship**- v  **Cbl**  suppresses apoptosis and differentiation, possibly through enhancement of  **Lyn** , Syk, and PI3 kinase activities and Bcl 2.  Ref: 11378270 Exp Hematol, 2001
- This phosphorylation was intact in Btk and  **Cbl**  deficient DT40 cells, but was drastically suppressed in  **Lyn** , Syk, or BCAP deficient DT40 cells.  Ref: 12627965 Biochemistry, 2003
**Neutral relationship**- Using yeast two hybrid analysis, we found that c  **Cbl**  directly couples  **Lyn**  to PI 3 kinase.  Ref: 12149655 Oncogene, 2002
- **Lyn**  associates with and phosphorylates a small set of molecules, including c  **Cbl** .  Ref: 12149655 Oncogene, 2002
- Through its Src homology 3 SH3 and SH2 domains, the Src kinase  **Lyn**  interacts with a small number of phosphoproteins, such as Shc,  **Cbl** , and Vav, which regulate cell cycle and the cytoskeleton.  Ref: 12456510 Blood, 2003
- Basal activities of  **Lyn** , Syk, and PI3 kinase were elevated in the v  **Cbl**  line.  Ref: 11378270 Exp Hematol, 2001

**Non-interactive relationship** (e.g. studied together, co-existance, homology, etc.)

- In this study, we demonstrate that HGF induces strong tyrosine phosphorylation of the proto oncogene product c  **Cbl**  in B cells and increases  **Cbl**  association with the Src family tyrosine kinases Fyn and  **Lyn** , as well as with phosphatidylinositol 3 kinase and CrkL.  Ref: 12244174 J Immunol, 2002
- Under our conditions, hydrogen peroxide induced PI3K and Akt activation was independent of  **Lyn** , Syk,  **Cbl** , BCAP, or Ras when each was eliminated individually either by mutation or by a specific inhibitor.  Ref: 12627965 Biochemistry, 2003
- In retinoic acid differentiated HL 60 cells, cross linking of FcgammaRs resulted in a marked increase in the tyrosine phosphorylation of FcgammaRIIa, p58  **lyn** , and p120 c  **cbl** , which was inhibited by a specific inhibitor of Src family protein tyrosine kinases.  Ref: 11698455 J Immunol, 2001
